# A Novel Quantitative Approach to Staging and Assessing Recovery from Type 1 Diabetes Mellitus: The Type 1 Diabetes Mellitus Metabolic Recovery Index

**DOI:** 10.3390/ijms21030992

**Published:** 2020-02-03

**Authors:** Tihamer Orban, Nara T. Orban, Heyam Jalahej, Piers E. F. Daubeney

**Affiliations:** DMNoMore/Phaim Ltd., Office 227, Chester House, 81-83 Fulham High Street, Fulham, London SW6 3JA, UK; nara.daubeney@dmnomore.com (N.T.O.); heyam.jalahej@dmnomore.com (H.J.); piers.daubeney@dmnomore.com (P.E.F.D.)

**Keywords:** type 1 diabetes mellitus, autoimmunity, recovery, metabolic assessment

## Abstract

Discovery of insulin in 1921 changed the lives of patients with type 1 diabetes (T1DM) forever. What had been a death sentence became a manageable, albeit chronic, disease. Insulin did not cure the disease, as it did not address the actual disease process, but instead treated its sequelae, namely elevated blood sugars. Importantly, insulin administration fails to ensure normoglycaemia. Even with the most sophisticated ‘near closed-loop’ methods, glucose homeostasis is not restored to normal. T1DM patients face complications, both short-term, such as hypo- and hyperglycaemia, and long-term, with increased glycosylation of proteins leading to eye, kidney, nervous system and other sequelae. These complications are associated with significant morbidity and mortality even after intensive insulin treatment. Nearly 100 years after the discovery of insulin, we continue to face the challenge of addressing the disease process itself, in order to fundamentally improve the life of these patients. There are major efforts to achieve just that: to completely arrest the autoimmune process destroying the insulin-producing cells in the pancreas, or at least significantly slow the process to blunt and delay short- and long-term complications. The aim of this Communication is to propose a novel assessment tool that would serve as a quantitative outcome measure by which therapies, short of clinical cure, may be compared and their true benefit to the treatment of diabetes assessed.

## 1. Introduction

Prior to the discovery of insulin [1], patients survived only a few years at most and the sole intervention available was enforced starvation. Insulin replacement therapy has transformed patients’ lives and prognoses. Nevertheless, even today with new sophisticated insulin replacement methodologies [2], they still face the daily challenges of abnormal blood sugars [3] and their short and long-term sequelae [4,5,6]. Cure still eludes clinicians and patients.

**Figure 1:**
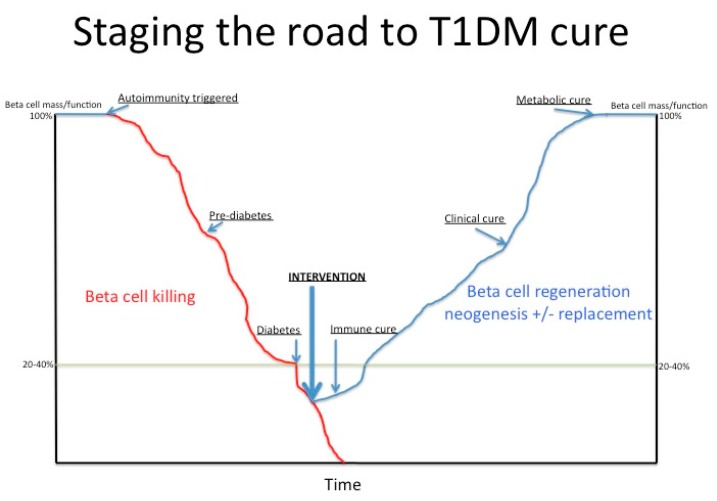
The road to clinical type 1 diabetes (T1DM) [7] and stages of recovery from the disease.

It may seem trivial to reflect on what is *cure* for type 1 diabetes (T1DM), but in fact it has several facets: immunological, clinical and metabolic, all of which require consideration and evaluation. Immunological cure implies that the auto-aggressive immune process is arrested; immune cells participating in the pathological process are either eliminated, incapacitated or fully suppressed. Clinical cure is signified by absence of the clinical symptoms of diabetes without any therapy, insulin or otherwise. This does not necessarily mean that the pancreatic beta cells are fully functional. Metabolic cure is a state where the full original beta cell mass and its function are fully restored. Metabolic cure encompasses both immunological and clinical cure (Figure 1).

## 2. Results and Discussion

The formula we propose for the Type 1 diabetes mellitus metabolic recovery index (DMMRI) is shown in Equation (1).

Δ HbA1c (P − T) + (5 × (1 − Δ Insulin dose (T − P)/1 − Δ Stimulated C-peptide AUC (T − P))(1)

P placebo, T treated.

Value of 5 is indicative of no effect for the intervention;

Values over 5 signify proportional improvement;

Values less than 5 signify proportional worsening of the metabolic status as a result of the intervention.

To assess immunological cure, all immune cells participating in beta cell destruction need to be identified and assays developed to measure their numbers, activities, functions and interactions. At the present time these ambitious aims are theoretical concepts and not yet developed into a practical methodology. The T1DM-specific pathological immune cells are not identified, and therefore comprehensive assessment of immunological cure remains elusive. Surrogate markers such as increased number and function of regulatory T cells or T cell cytokine profile changes are very useful markers but fall short of fully and accurately assessing immunological cure.

Clinical cure is much easier to define: no clinical symptoms, normal blood sugars (normal HbA1c) and no treatment of any sort, including insulin. This does not necessarily mean that beta cell health is fully restored. The beta cell function could be anywhere between ~50% and 100% as it is in the so-called pre-diabetes state. One might also call it the ‘post-diabetes state’.

Documenting metabolic cure is also relatively straightforward. Normal serum insulin levels reflect restoration of normal endogenous insulin production. A normal measurement of the first phase insulin response to glucose is the closest we can get to record restored beta cell function.

The last 50 years have seen increasing and concerted efforts to intercept T1DM autoimmunity, with two main approaches to treatment: immune cell targeted therapies, or antigen focused therapies. Each of these modalities to date falls short of achieving clinical cure let alone metabolic cure.

The critical shortcoming of the immune cell targeted therapies is the lack of identification of the T1DM specific immune cell(s) participating in and driving the autoimmunity. The current understanding is that existing T1DM specific CD4+ T cells [8], uncontrolled by the regulatory T cell [9], get activated, and antigen presenting cells (APCs) [10], CD8+ T cells [11] and likely other parts of the immune system join the orchestra of autoimmune destruction of the pancreatic beta cells. Without knowing the identity of the T1DM specific T cells and other immune cells participating in the expanding network of autoimmunity as the disease progresses, it is impossible to develop T1DM specific immune suppressive therapies. Some trials have used blanket immune suppression such as ATG (anti-thymocyte globulin) [12], while others employed more T-cell or APC (B-cell) specific agents such as antiCD3 [13], CTLA4-Ig [14] or antiCD20 [15]. Some such as antiCD3 and CTLA4-Ig managed to alter, but not stop, the course of the autoimmunity with some clinically important benefits and insights.

Antigen focused therapies have not fared much better. The main hindrance is twofold, namely identification of the ‘right’ antigen(s) and finding the ‘right’ delivery system. There are good arguments to support a central role for insulin or related antigen(s) in the initiation of the autoimmunity. One is that the destruction is restricted and specific to the insulin-producing beta cell in the pancreatic islet. This is complicated by the fact that there is epitope, as well as antigen-spreading as the disease advances [16]. One could envisage that unless an intervention in this process takes place at a very early stage of the disease, there may be a need to employ combinations of key antigens, perhaps in an individualised fashion. Oral insulin has been tried in a prevention setting [17] and post hoc analysis indicated some preventive effect in a subgroup [17]. GAD in alum in T1DM patients showed modest beneficial effects in a recent metanalysis [18]. Insulin B-chain in incomplete Freund’s adjuvant (IFA) in a phase 1 clinical trial showed a desirable immune effect by increasing insulin B-chain specific regulatory T cells [19]. Finding a delivery system powerful and targeted enough to drive the immune system back to immune tolerance is an elementary component of an antigen-based approach. IFA or similarly powerful adjuvant(s) may be the answer. Deactivating the auto-aggressive immune cells by reducing their number and/or function and thus restoring the balance and tolerance is the key. The aim is to mirror the thymic education of immune cells in the peripheral circulation.

Neither immune cell targeted, nor antigen focused therapeutic interventions resulted in clinical cure of T1DM. Some successfully altered the pathology but fell short of arresting the autoimmunity. Nevertheless, they have resulted in some beneficial clinical outcomes. The challenge is how to best evaluate these partial, but very important results and how to compare success in trials with varying methodology, aiding the direction of further trials.

The key metabolic feature of the disease is the destruction of insulin production. It would seem logical to place the measurement of self-insulin production in the centre of assessment of success. This on its own is insufficient and needs to be combined with critical clinical and laboratory parameters. Currently the best method to measure self-insulin production is serum C-peptide. Other clinically important parameters are the metabolic effect of self-insulin production, namely blood sugar levels (HbA1c), amount of exogenously delivered insulin, frequency of hypoglycaemic and hyperglycaemic events (DKA). These parameters are interconnected; one might say they are in ‘linkage disequilibrium’. The linkage between C-peptide, HbA1c and insulin dose is probably tighter than linkage of this group with the other parameters (hypoglycaemic episodes, major hyperglycaemic episodes/DKA). Better understanding of the delicate relationships between surrogate markers of diabetes control will aid the comparison of new treatment modalities and by extension, the development of new therapies.

In the prediabetes setting there have been efforts to better assess the metabolic decline heading to T1DM. Measurement of changes in stimulated C-peptide over time alone [20,21] or in combination with HbA1c [22] showed promise in improving prediction of the onset of T1DM beyond the customary autoantibody measurements. As of today, there has been no attempt to gauge the dynamic of the metabolic deterioration or improvement in a comprehensive manner in patients with T1DM.

Most of the clinical trials in T1DM use stimulated C-peptide as the primary outcome measure with insulin usage and HbA1c as secondary measures of effectiveness, but not together in combination. It is very likely that a trial where C-peptide preservation also manifests better HbA1c will have a bigger impact on the patient’s short and long-term health. If these two changes occur as well as lower insulin usage, it may imply even more benefit from the therapy employed. It would seem desirable to combine these parameters into a formula.

The potential utility of our proposed new index (Table 1) is to quantify the degree of benefit of the interventions, short of clinical cure. The maintenance or increase of the positive index (DMMRI > 5) over time could indicate immune cure, whereas improved but subsequent decline over time would indicate beneficial but temporary alteration of the autoimmune process. The index could also indicate negative effects of the interventions accelerating autoimmunity and thus worsening the metabolic status (DMMRI < 5). The other potential utility of the index is to help in selecting the best, most promising combination therapies. Beyond considering the potential additive and/or synergistic effects of different interventions, this index could help to make the best selection by choosing the therapies with the highest indices.

There are a number of caveats to this index. Our index is only applicable in placebo controlled interventional clinical trial settings. Different trials may use different T1DM patient populations with for example different duration of intervention, etc. This is true for the three trials where the index has been tested. Nevertheless, the derived indices seem to support the utility of this approach. The two successful trials, rituximab and abatacept, yielded indices over five. The abatacept index is higher than for rituximab perhaps indicating more benefit for the patients. The GAD vaccination trial was deemed ineffective and this is supported by a DMMRI of less than five. In the future, further testing will be needed to gauge the utility of this index.

Efforts to cure this dreadful disease are continuing in earnest with new trials underway and more planned. A better understanding of the relative merits of these new interventions in T1DM is much needed to guide strategies and designs for future drugs and combination therapies. Here we have described a novel index that allows comparison of such studies that will assist present and future endeavours to ameliorate and ultimately cure T1DM, our ultimate aim.

## 3. Materials and Methods

George Eisenbarth, in 1984 [7], described the phases of the autoimmune process in type 1 diabetes. This gave us a new insight into natural disease progression. We now complement this with a novel conceptual review of the recovery phases from the autoimmune destruction. The last few decades of the 20th century were focussed on better understanding of the disease process itself. Now that new, potentially curative interventions are the focus of research, there is an urgent need for a tool that quantifies and predicts stages of recovery from type 1 diabetes.

Our report provides a novel conceptual approach (summarised in Figure 1), to better characterise and stage treatments and interventions that set out to alter type 1 diabetes mellitus autoimmunity in a beneficial way. There is a clear need to objectively and quantitatively assess the progress, or the lack of it, in clinical interventional trials. Importantly, there is also a need to reliably compare interventions to find those with the biggest beneficial impacts and potentially combine them for synergistic effects.

In developing our DMMRI (Equation (1)) we used outcome data from three published clinical trials [14,15,23] that reported stimulated C-peptide area under the curve, HbA1c and insulin doses. We chose these three clinical trials as they are the most recent and comprehensive, have rather similar clinical protocol designs and have all these parameters tested. Our index is based on the concept that higher C peptide is linked to lower HbA1c and lower insulin usage. Combining these three parameters into one formula provides the best method of assessing overall outcomes.

To the best of our knowledge, this is the first attempt at combining these three interlinked clinical parameters to accurately describe the level and rate of metabolic change in the disease process. This should provide a more comprehensive measure of the efficacy of interventions aimed at altering the clinical course of type 1 diabetes mellitus. It is also suitable to accurately predict the rate of decline in metabolic status in ongoing disease.

The data collection and analysis were reviewed and approved internally by the DMNoMore/Phaim Ltd. Ethics Board.

## 4. Conclusions

In this report we propose a novel conceptual approach to characterise and stage recovery from autoimmune destruction in type 1 diabetes mellitus. This complements the stages of decline described by Eisenbarth. This staging helps to classify the efficacy of any intervention aimed at arresting damage to insulin producing beta cells. Furthermore, in this paper we propose a novel assessment tool, DMMRI, that should now serve as a composite quantitative metabolic outcome measure by which therapies may be compared and the true benefit of diabetes treatment assessed.

## Figures and Tables

**Table 1:** Type 1 diabetes mellitus metabolic recovery index (DMMRI) for rituximab [15], abatacept (CTLA4-Ig) [14] and GAD [23].

		Rituximab [15] (Age: 8–40 Years; Duration 1 Year)	Abatacept [14] (Age: 6–45 Years; Duration 2 Years)	GAD Vaccine [23] (Age: 3–45 Years; Duration 1 Year)
HbA1c (%)	Placebo	7.0	7.9	7.1
	Treated	6.76	7.2	7.1
Insulin dose (U/kg/day)	Placebo	0.48	0.65	0.56
	Treated	0.39	0.61	0.63
Stimulated C-peptide AUC (nmol/L)	Placebo	0.47	0.238	0.413
	Treated	0.56	0.378	0.412
DMMRI		**6.22**	**6.74**	**4.69**
